# Supplementary material for: Atomic Arrangements of Graphene-like ZnO
Source: Nanomaterials (Basel). 2021 Jul 14;11(7):1833. doi: 10.3390/nano11071833 (PMC8308407; doi:10.3390/nano11071833)
Supplement: Supplementary file 1 [file nanomaterials-11-01833-s001.zip › nanomaterials-1287373-supplementary.pdf]

Supplementary Materials

Atomic Arrangements of Graphene-Like ZnO

Jong Chan Yoon [1,2], Zonghoon Lee [1,2] and Gyeong Hee Ryu [3,*]

[1] Department of Materials Science and Engineering, Ulsan National Institute of Science and Technology (UNIST), Ulsan 44919, Korea; yjc1526@unist.ac.kr (J.C.Y.); zhlee@unist.ac.kr (Z.L.)
[2] Center for Multidimensional Carbon Materials, Institute for Basic Science (IBS), Ulsan 44919, Korea
[3] School of Materials Science and Engineering, Gyeongsang National University, Jinju 52828, Korea
* Correspondence: gh.ryu@gnu.ac.kr

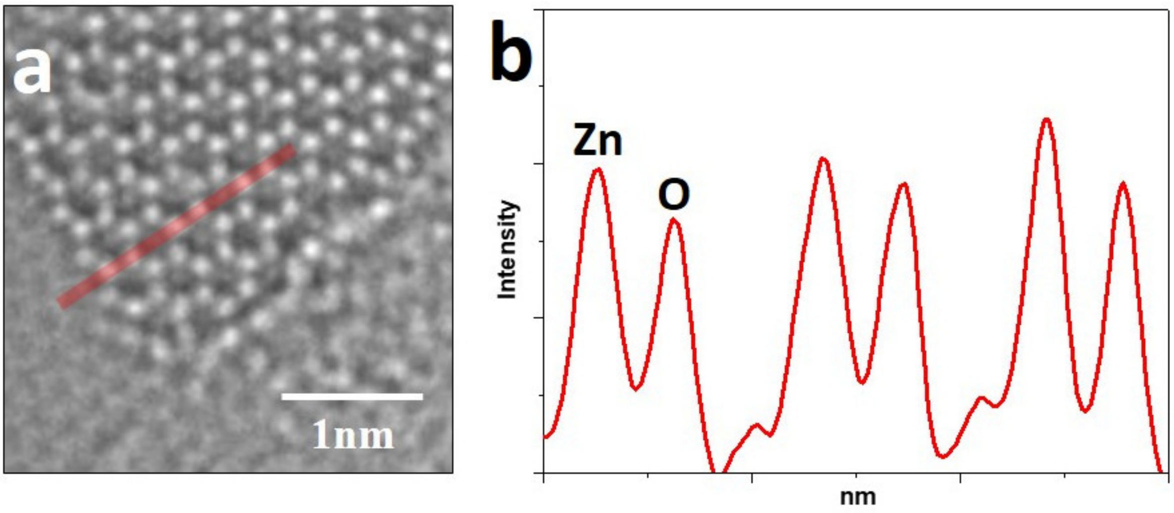

Figure S1. (**a**) Raw image of Figure 3(e). (**b**) Intensity line profiles acquired from the red line in (a). This indicates that the ZnO flake has O-terminated ZZ configuration.

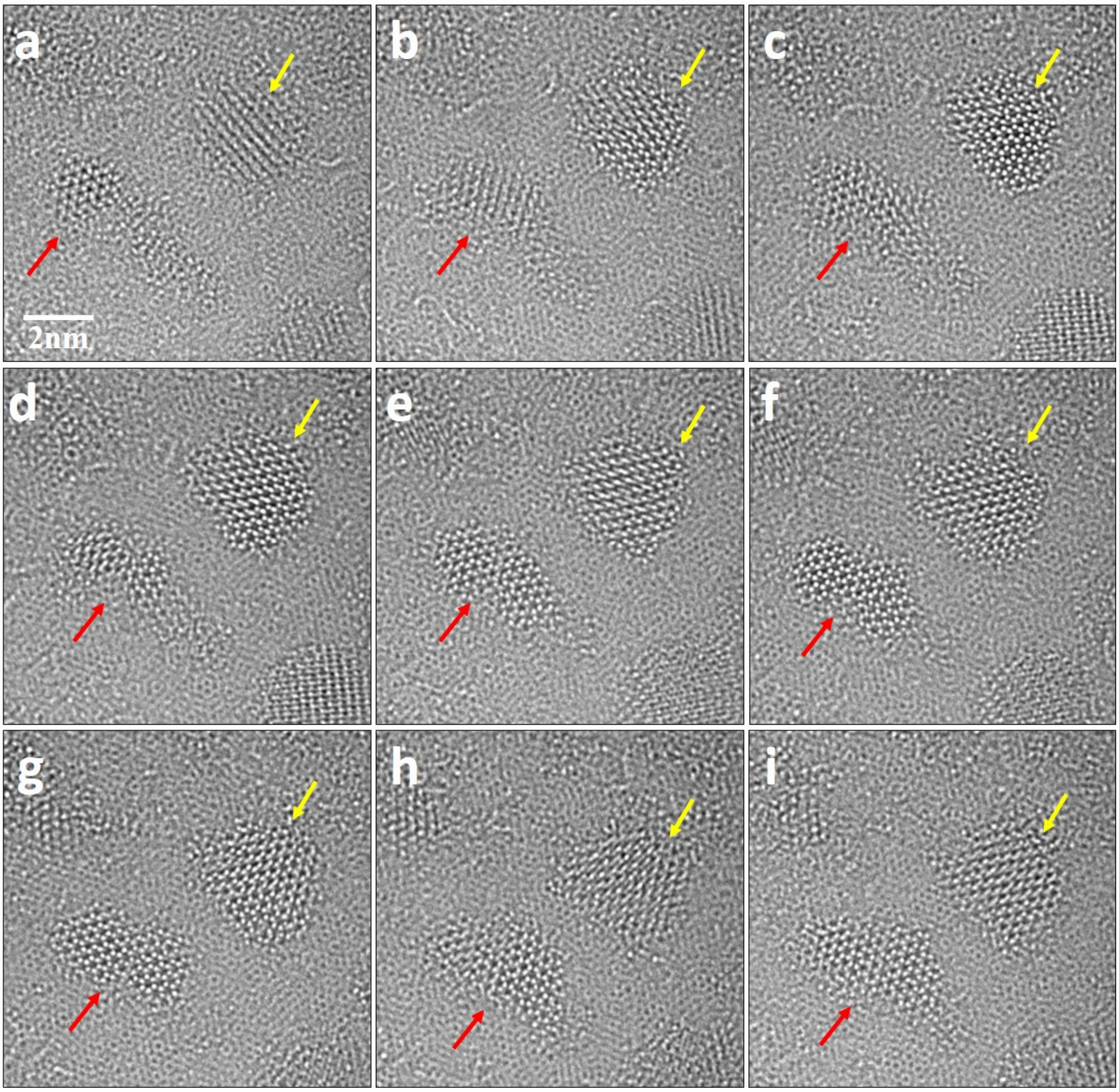

Figure S2. Atomic g-ZnO flakes on the graphene. (**a**–**i**) ACTEM images showing the flakes atomically driven under electron beam irradiation.

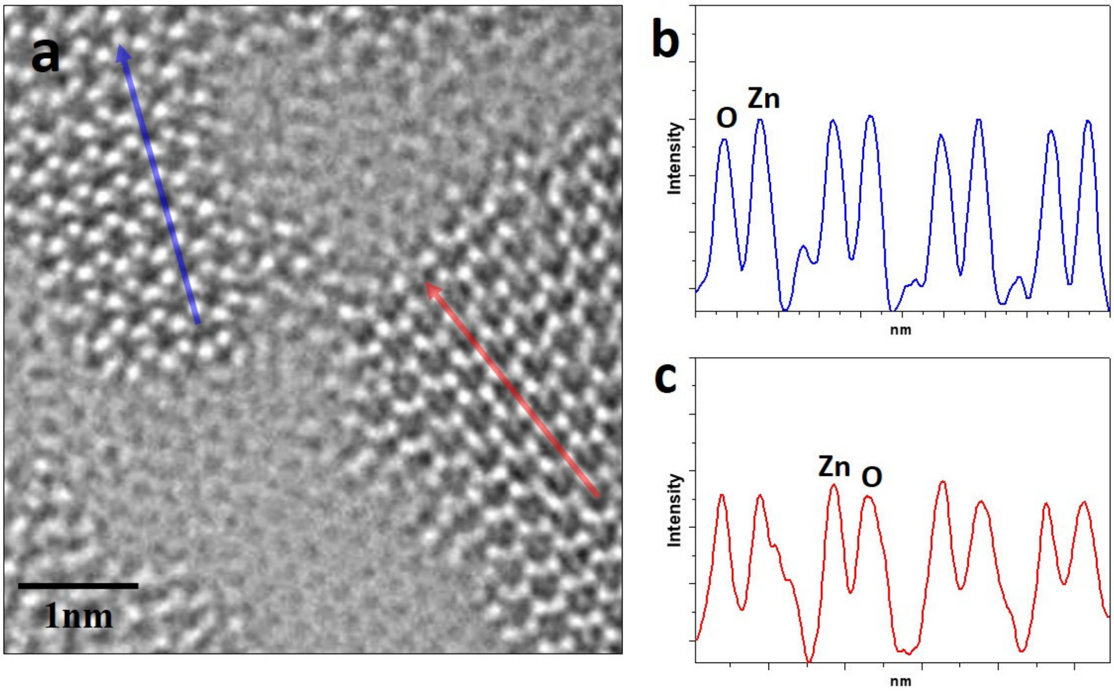

Figure S3. (**a**) Raw image of Figure 5(a). (**b**) and (**c**) Intensity line profiles acquired from blue and red arrows in (a).

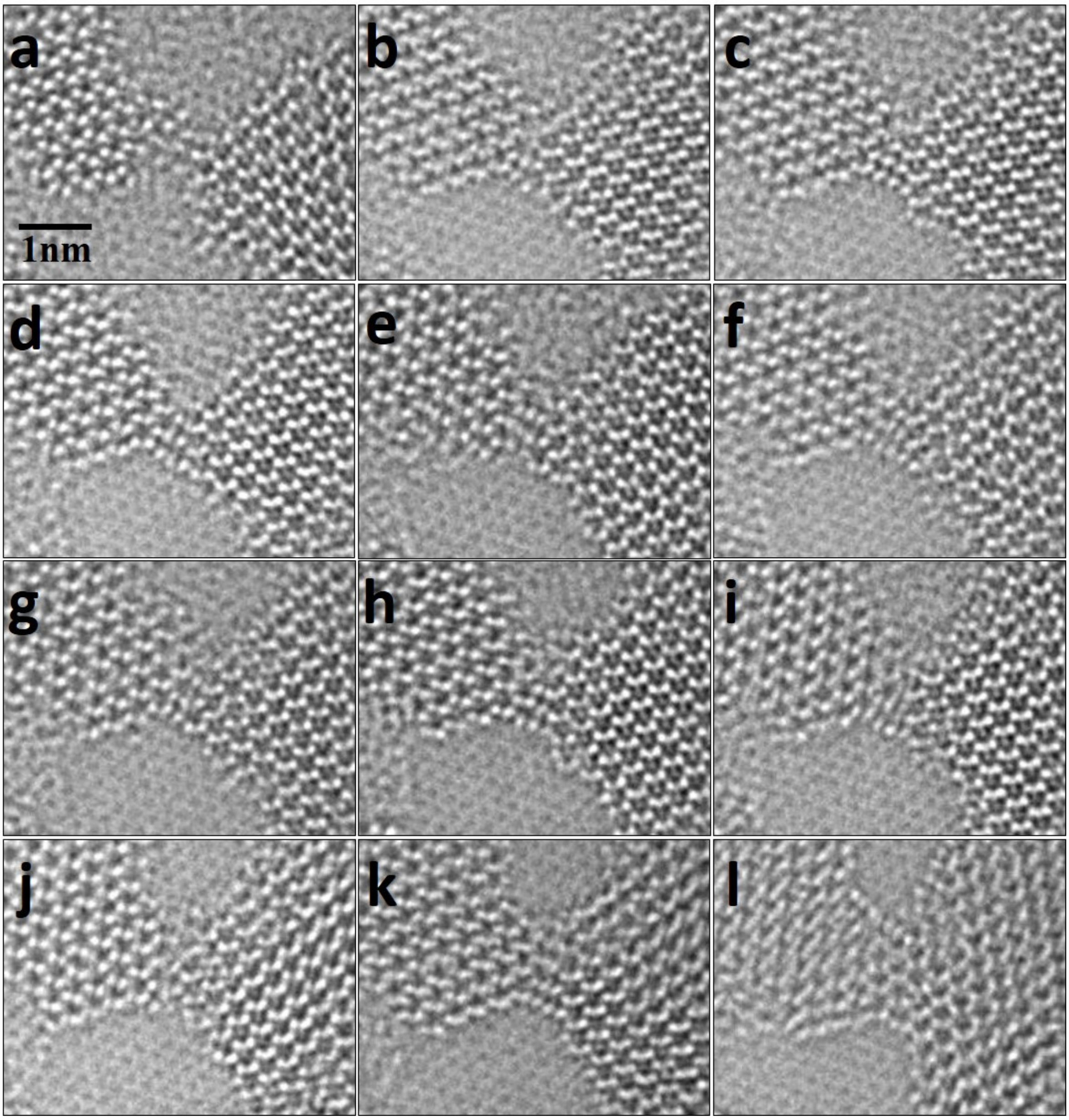

Figure S4. ACTEM images showing the full processes of the merging between the g-ZnO flakes. .
